# Navigating Adherence: Unraveling Factors Shaping Opioid Substitution Therapy Compliance

**DOI:** 10.7759/cureus.51577

**Published:** 2024-01-03

**Authors:** Anupam S Yadav, Ashutosh Kumar, Sonali Singh, Tejpal Singh

**Affiliations:** 1 Department of Psychiatry, Ganesh Shankar Vidyarthi Memorial (GSVM) Medical College, Kanpur, IND; 2 Department of Psychiatry, Sarojini Naidu Medical College, Agra, IND; 3 Department of Pediatrics, King George's Medical University, Lucknow, IND; 4 Department of Medicine, Sarojini Naidu Medical College, Agra, IND

**Keywords:** buprenorphine, ost, opioid, follow-up, drop-out, addiction

## Abstract

Background and objective

In drug-deaddiction programs, dropout is a major problem in any drug de-addiction program, as dependence is a chronic illness known to relapse frequently. Understanding factors that predict dropout can help design targeted interventions to promote follow-up. This study aimed to assess the various sociodemographic characteristics of opioid-dependent subjects on buprenorphine maintenance treatment and dropping out at or before the three-month follow-up period.

Method

In this study, the sociodemographic characteristics and quality of life (QOL) of 34 opioid-dependent subjects (males, 32, 94%; females, 2, 6%) on the day of their enrolment in an opioid substitution therapy (OST) center were assessed, and a comparison of sociodemographic and drug use pattern was made between those who followed up and those who dropped out by the end of three months.

Results

Statistical analysis of the various sociodemographic characteristics using appropriate tests yielded that predictors of good follow-up are younger age (*F* = 4.57907, *P* = 0.04008), better education (*F* = 5.07221, *P* = 0.031305), and being part of a nuclear family. Longer follow-up was associated with shorter opioid intake duration (*F* = 8.58908, *P* = 0.006195). Better social relationships, as evidenced by the social relationship domain score of QOL, predicted longer follow-up (*F* = 8.58908, *P* = 0.006195). Other characteristics analyzed did not yield significant associations.

Conclusions

The study unveils the complexity of opioid addiction recovery, revealing the interplay of age, education, family, addiction duration, and support, shaping one's resilience in recovery.

## Introduction

India’s peculiar location between the two largest sources of illicit drugs in South Asia - the Golden Crescent (Pakistan, Afghanistan, and Iran) on the northwest and the infamous Golden Triangle (Myanmar, Thailand, and Laos) on the northeast - predisposes it to illicit drug trafficking as well as drug use, with opioids being one of the most prominent ones. Hence, it is not surprising to note that India has twice the global average prevalence of illicit opiate consumption. India has approximately 7.7 million individuals with opioid use disorders. More than a quarter (26%) of patients in drug addiction treatment centers identify opioids as their primary drug of abuse [1]. Heroin is the most prevalent opioid used across the nation, with pharmaceutical opioids (injection buprenorphine and injection pentazocine) and opium ((or its variants like poppy husk known as doda/phukki) following closely behind in terms of common usage. Opioid dependence is a chronic condition that impacts not only physical health but also various aspects of life, thereby influencing overall quality of life (QOL) [2]. The available pharmacological options for detoxification and long-term treatment of opioid dependence include agonists (methadone, buprenorphine, and L-alpha acetyl methanol [LAAM]), antagonists (e.g., naltrexone), and non-opioid agents (e.g., alpha-2 adrenergic) [3]. The current evidence from the West supports the efficacy of opioid substitution therapy (OST) in improving treatment retention, reducing illicit opioid use, enhancing QOL, and lowering the incidence of human immunodeficiency virus (HIV) and hepatitis C virus (HCV) infections [4-6]. OST is endorsed by the Joint United Nations Program on HIV/AIDS (UNAIDS), the United Nations Office on Drugs and Crime (UNODC), and the World Health Organization (WHO) for injecting drug use (IDU) programs that maximize impact on HIV prevention and treatment [7]. Opioid dependence is a chronic condition affecting not just physical health but also other domains of life and, thus, overall QOL. To address this threat, both governmental and non-governmental organizations conduct de-addiction programs. De-addiction programs span an extended period, and discontinuation during the program presents a significant issue, ultimately leading to relapse and treatment failure [8,9].

While the effectiveness of OST is well-documented in middle- and high-income countries, evidence from low-income countries remains sparse. This discrepancy underscores an urgent need for targeted research in these regions. Such studies are crucial for understanding the unique socioeconomic and health infrastructure challenges that might influence OST effectiveness and adherence in low-income settings [5,10,11,12]. They are also vital in identifying the factors that affect retention in these programs, which could be significantly different due to various constraints and cultural contexts. This research would not only inform policy-making and efficient resource distribution but also have broader public health implications. It is especially crucial for global health strategies targeting the control of infectious diseases prevalent among individuals who inject drugs. Systematic and focused investigations are essential in low-income countries to gain insights into the dynamics of OST programs, ultimately guiding strategies to enhance their effectiveness and impact on public health.

Thus, this study compares the sociodemographic characteristics and QOL of opioid-dependent subjects at the beginning of buprenorphine maintenance treatment between those who follow up at the end of three months and those who drop out earlier.

## Materials and methods

Ethical approval from the Institutional Ethics Committee was duly taken before the initiation of the study. In this clinical study, a total of 44 patients were enrolled in the OST center at a tertiary care center in North India, during the study period from October 2020 to November 2021. The sample size was curtailed considering the recruitment challenges, dropouts, and resource limitations. Out of these, 34 patients fulfilled the inclusion criteria and gave consent to participate in the study. Inclusion criteria included individuals aged 18 years or older, with a minimum of five years of opioid use, self-reporting illicit opioid use in the past 30 days, experiencing two or more unsuccessful attempts at abstinence, meeting criteria for opioid dependence as per the International Classification of Diseases, 10th edition (ICD-10), expressing willingness to take buprenorphine, and providing consent to participate in the study. Patients with serious medical conditions, such as acute respiratory failure, acute hepatic disease, delirium tremens, current alcohol dependence, female users who were pregnant or breastfeeding, patients with known hypersensitivity to buprenorphine, and those with major psychiatric or physical illnesses preventing cooperation for an interview, were excluded.

Sociodemographic and clinical data were collected, and then patients were assessed with the Schedules for Clinical Assessment in Neuropsychiatry (SCAN) to assess for any mental illness. SCAN is a WHO-developed, semistructured, standardized clinical interview with patient cross-examination. The Clinical Opiate Withdrawal Scale (COWS) was applied to assess withdrawal symptoms in the patient. COWS is an 11-item clinician-administered scale. The summed score determines the severity of opiate withdrawal and level of physical dependence [13]. The QOL of patients was then assessed with the World Health Organization Quality-of-Life Instrument (WHOQOL-BREF). WHOQOL-BREF has 26 items, which measure the broad domains of physical health, psychological health, social relationships, and environment. The four domains are scored, labeled, and scaled from 0 to 100 [14,15]. Data about follow-up after three months of enrollment was collected from the OST center. A comparison of sociodemographic characteristics and QOL at baseline was made between those who followed up and those who dropped out by the end of three months. Utilizing IBM SPSS Statistics for Windows, Version 28.0 (IBM Corp., Armonk, NY) for statistical analysis, descriptive statistics were employed in the form of mean ± standard deviation, median, and absolute frequency. Statistically, a correlation between the findings and a P-value of <0.05 indicates significance.

## Results

As shown in Table 1, the study sample comprised 32 (94%) males and 2 (6%) females. The mean age of the study population at enrollment was 35.3 ± 11 years. Twenty (59%) patients of the study sample were married, 11 (32%) were unmarried, and 3 (9%) were married but single, i.e., widowed, divorced, or separated. Maximum number of participants were from urban areas (74%). The educational status of the study population was low for the majority of the patients: 10 (29%) were illiterate; 8 (24%) were educated up to primary school, that is, up to the fifth grade; and 7 (21%) attended middle school, i.e., up to the eighth grade.

**Table 1:** Sociodemographic characteristics.

		Dropout (*N *= 11)	Follow-up (*N *= 23)	Total (*N *= 34)
Sex	Male	11 (34%)	21 (66%)	32 (94%)
	Female	0 (0%)	2 (100%)	2 (6%)
Marital status	Married	6 (30%)	14 (70%)	20 (59%)
	Single	5 (36%)	9 (64%)	14 (41%)
Residence	Rural	2 (22%)	7 (78%)	9 (27%)
	Urban	9 (36%)	16 (64%)	25 (73%)
Living arrangement	With family	10 (40%)	15 (60%)	25 (73%)
	Away from family (hostel, barracks, etc.)	1 (11%)	8 (89%)	9 (27%)
Family type	Nuclear	2 (13%)	14 (87%)	16 (47%)
	Joint	9 (50%)	9 (50%)	18 (53%)
Usual route of intake	Parenteral	10 (40%)	15 (60%)	25 (73%)
	Others	1 (11%)	8 (89%)	9 (27%)
HIV status	Positive	1 (20%)	4 (80%)	5 (15%)
	Negative	10 (35%)	19 (65%)	29 (85%)
Education	Illiterate	5 (45%)	5 (22%)	10 (29%)
	Primary school certificate	4 (36%)	6 (26%)	10 (29%)
	Middle school certificate	2 (18%)	5 (22%)	7 (21%)
	High school certificate	0 (0%)	2 (9%)	2 (6%)
	Intermediate/post-high school diploma	0 (0%)	4 (17%)	4 (12%)
	Graduate or postgraduate	0 (0%)	0 (0%)	0 (0%)
	Profession or honors	0 (0%)	1 (4%)	1 (3%)
Employment status	Professional	0 (0%)	1 (4%)	1 (3%)
	Skilled worker	2 (18%)	7 (30%)	9 (27%)
	Unskilled worker	6 (54%)	9 (39%)	15 (44%)
	Student	0 (0%)	2 (9%)	2 (6%)
	Unemployed	1 (9%)	4 (17%)	5 (15%)
	Retired	2 (18%)	0 (0%)	2 (2%)

Five (15%) out of the 34 subjects were HIV-positive at the time of enrollment. We see that 19 (56%) subjects had monthly family income in the range of ₹3,908-11,707, 7 (21%) earned ₹11,708-19,515, and 4 (12%) earned ≤₹3,907, i.e., the majority belonged to the low-income group (Tables 1-2). The majority of subjects were unskilled workers (15, 44%), followed by the unemployed.

**Table 2:** Mean value of demographic and drug use characteristics.

	Dropout (*N *= 11)	Follow-up (*N *= 23)	Total (*N *= 34)
Age (years)	41.23 ± 13.24	32.47 ± 10.15	35.3 ± 11.8
Education (years)	7.39 ± 4.97	3.37 ± 3.31	6.35 ± 4.94
Family income per month (₹)	10,254 ± 7,893	18,500 ± 27,152	15,832 ± 22,929
Age at first use of primary drug (years)	18.55 ± 4.74	18.96 ± 6.41	18.82 ± 5.85
Duration of opioid intake (years)	11.59 ± 3.92	8.52 ± 2.21	9.51 ± 3.16

Pharmaceutical opioids such as injection buprenorphine, injection pentazocine, and other opioids such as phukki, doda, etc., were the most commonly used substances. The majority (77%) of subjects used the primary drug usually through an intramuscular or intravenous route. The mean duration of opioid intake was 9.51 ± 3.16 years (Table 2).

A comparison was made between those who followed up and those who dropped out by the end of three months (Tables 3-4). Younger age (*F* = 4.57907, *P* = 0.04008) and a greater number of years of education (*F*= 4.2826, *P *= 0.046659) had significant associations with treatment retention. Subjects who belonged to nuclear families showed longer duration of follow-up.

**Table 3:** One-way analysis of variance (ANOVA) test for significance of sociodemographic characteristics.

Variable	Group	Mean	Standard deviation	*F*-ratio	*P*-value	Significance at *P *< 0.05
Age	Follow-up (*N *= 23)	32.47	10.15	4.57907	0.040089	Significant
	Dropout (*N *= 11)	41.27	13.24			
Family income	Follow-up (*N *= 23)	18,500	27,152	0.96117	0.334248	Not significant
	Dropout (*N *= 11)	10,254	7,893			
Education (in years)	Follow-up (*N *= 23)	7.39	4.97	6.17923	0.018339	Significant
	Dropout (*N *= 11)	3.27	3.31			
Employment status	Follow-up (*N *= 23)	3.04	1.15	1.20766	0.279996	Not significant
	Dropout (*N *= 11)	3.54	1.43			
Duration of opioid intake	Follow-up (*N *= 23)	8.52	2.21	8.58908	0.006195	Significant
	Dropout (*N *= 11)	11.59	3.92			
Age at first use	Follow-up (*N *= 23)	18.96	6.41	0.03562	0.8515	Not significant

**Table 4:** Fisher's exact test for significance of sociodemographic characteristics, drug use, and HIV status.

Variables				Statistical value	Significance at *P* < 0.05
Sociodemographic characteristics					
Sex		Male	Female	0.549	Not significant
	Follow-up (*N *= 23)	21 (66%)	2 (100%)		
	Dropout (*N *= 11)	11 (34%)	0 (0%)		
Residential status		Rural	Urban	0.6824	Not significant
	Follow-up (*N *= 23)	7 (78%)	16 (64%)		
	Dropout (*N *= 11)	2 (22%)	9 (36%)		
Marital status		Married	Single	1	Not significant
	Follow-up (*N *= 23)	14 (70%)	9 (64%)		
	Dropout (*N *= 11)	6 (30%)	5 (36%)		
Family type		Nuclear	Joint	0.0296	Significant
	Follow-up (*N *= 23)	14 (87%)	9 (50%)		
	Dropout (*N *= 11)	2 (13%)	9 (50%)		
Living arrangement		With family	Away	0.2137	Not significant
	Follow-up (*N *= 23)	15 (60%)	8 (89%)		
	Dropout (*N *= 11)	10 (40%)	1 (11%)		
Drug use and HIV status					
Route of intake		Parenteral	Others	0.2137	Not significant
	Follow-up (*N *= 23)	15 (60%)	8 (89%)		
	Dropout (*N *= 11)	10 (40%)	1 (11%)		
HIV status		Negative	Positive	0.6499	Not significant
	Follow-up (*N *= 23)	19 (65%)	4 (80%)		
	Dropout (*N *= 11)	10 (35%)	1 (20%)		

Other sociodemographic characteristics such as family income, sex, residential status, marital status, family type, living arrangement, education, employment status, and HIV status of both groups did not yield any significant difference.

Statistical analysis using the one-way analysis of variance (ANOVA) test for significance revealed that a lesser duration of opioid intake was associated with longer follow-up (*F* = 8.58908, *P* = 0.006195). No significant difference in age at first use or route of intake was found between the two groups.

Better social relationships, as evidenced by the social relationship domain score of QOL, predicted longer follow-up (*F* = 8.58908, *P* = 0.006195) (Table 5). Other domains of WHOQOL analyzed did not yield a significant difference.

**Table 5:** One-way analysis of variance (ANOVA) test for significance of the World Health Organization Quality-of-Life (WHOQOL-BREF) scores.

WHOQOL-BREF scores						
QOL domains	Sample group	Mean score	Standard deviation	*F*-ratio	*P*-value	Significance at *P *< 0.05
Physical health domain	Follow-up (*N *= 23)	19.78	6.08	1.72837	0.197964	Not significant
	Dropout (*N *= 11)	16.73	6.87			
Social relationships domain	Follow-up (*N *= 23)	66.65	13.52	13.89074	0.000749	Significant
	Dropout (*N *= 11)	49.46	10.21			
Psychological domain	Follow-up (*N *= 23)	24.26	6.97	0.71814	0.403048	Not significant
	Dropout (*N *= 11)	22.27	4.93			
Environmental domain	Follow-up (*N *= 23)	11.26	6.35	0.17231	0.680838	Not significant
	Dropout (*N *= 11)	12.18	5.32			

## Discussion

The study provides invaluable insights into the multifaceted determinants of success for individuals on the path to recovery from opioid addiction. It conducts a comprehensive examination of the intricate interplay between demographics, personal traits, addiction history, and the support systems that collectively shape the outcomes of efforts to overcome opioid dependence.

In the study conducted by Dhawan and Chopra spanning multiple regions across India and carried out under the purview of the National Drug Dependence Treatment Centre (NDDTC) at the All India Institute of Medical Sciences (AIIMS), New Delhi, an uneven gender distribution was observed within the sample comprising 231 individuals dependent on opioids. This distribution exhibited a conspicuous skew, with 5.2% of subjects being female and 94.8% male [16]. In our study, there were 32 (94%) males and 2 (6%) females. This small difference in gender distribution could potentially be attributed to various factors, such as geographical location, the availability of treatment facilities, and cultural or societal influences on help-seeking behavior among individuals dealing with opioid dependence.

Furthermore, when comparing our study to prior research, variations in the age of subjects emerge. In the study conducted by Habrat et al., the mean age of participants was documented as 33.9 ± 5.95 years, while Kapoor et al. in 2015 reported a mean age of 39.11 ± 8.31 years [17,18]. In our investigation, the mean age of the study population at the time of enrollment was 35.3 ± 11 years. These disparities in age distributions may be indicative of geographical variations in opioid dependence patterns.

The marital status of participants also stands as a noteworthy demographic factor. In our study, 20 (59%) patients were married, 11 (32%) were unmarried, and 3 (9%) were categorized as married but single, including widowed, divorced, or separated individuals. This distribution deviates from the findings reported in the study by Dhawan et al., where the proportions were 54% married, 34% unmarried, and 12% in the married but single category. This variation could potentially be attributed to regional disparities in marriage and family structures [16].

According to the Punjab opioid survey of 2014, 56% of opioid-dependent individuals in the state belonged to rural areas [19]. However, in our study, the majority of participants were from urban areas (74%). This difference is likely because the study site is located in an urban area, and its clients come from nearby places.

In the study by Wang et al., a notable 9.5% of participants were identified as HIV-positive during enrollment [20]. Our study similarly reflects a substantial prevalence of HIV among our subjects, with 5 out of the 34 participants (15%) testing positive at the time of their enrollment. This observation underscores the ongoing public health challenge of HIV co-occurrence within the context of opioid dependence, demanding comprehensive attention and interventions at the intersection of substance use and infectious diseases.

Income levels in the study population indicate a preponderance of individuals falling into the low-income group, keeping in mind the international extreme poverty line of $2.15 per person per day as per the World Bank. The majority (56%) reported a family income within the range of ₹3,908-11,707 (47-140 USD), with 21% earning ₹11,708-19,515 (140-234 USD) and 12% earning ≤₹3,907 (47 USD). This aligns with the findings of Kapoor et al. [18], where a significant portion of participants had a monthly income of less than ₹3500. These income disparities highlight socioeconomic factors that may significantly impact the ability to access and afford addiction treatment services.

Concerning educational status, the majority of participants in our study exhibited low levels of education: 10 (29%) were illiterate, 8 (24%) had completed primary school (up to the fifth grade, i.e., five years of formal education), and 7 (21%) had attended middle school (up to the eighth grade, i.e., 13 years of formal education). This finding highlights the prevalence of limited educational attainment among individuals seeking treatment for opioid dependence. The potential implications of low educational levels on access to and comprehension of addiction treatment programs warrant careful consideration.

The recent study revealed a predominant abuse of pharmaceutical opioids such as injectable buprenorphine and pentazocine, along with other illicit substances like phukki and doda. These findings contrast with the 2019 survey report by the Ministry of Social Justice and Empowerment [2]. This divergence likely stems from effective law enforcement strategies that have primarily restricted the availability of opioids other than those obtained pharmaceutically.

In the study by Dhawan et al., 48% of the drugs were used by chasing, 44% were intravenous, and 8% were smoked [16]. In the present study, the majority of cases (77%) involved subjects typically administering their primary drug through intramuscular or intravenous methods.

Remarkably, a significant majority, accounting for 77% of the study's participants, revealed that their preferred mode of drug consumption primarily involved intramuscular or intravenous routes. The utilization of intramuscular and intravenous methods often indicates a more advanced stage of addiction, potentially associated with increased physical and psychological dependence on opioids. Moreover, the choice of administration routes can significantly impact the risk of complications, including infections and overdose, heightening the urgency of tailored harm reduction strategies and treatment interventions for this vulnerable population. It highlights the pressing need for multifaceted approaches that encompass not only addiction treatment but also harm reduction initiatives to address the diverse challenges posed by opioid dependence.

The mean duration of opioid intake in this study was approximately 9.12 ± 2.85 years as compared to 8.4 ± 5.1 years in the study by Dhawan et al. [16]. These findings shed light on the prevalence of injection formulation of opioids and the chronic nature of opioid dependence within the studied population.

Subsequent analysis focused on factors exerting influence on treatment retention. Notably, younger age and more years of education were found to be significantly associated with extended treatment retention. The relationship between younger age, education, and treatment retention has also been explored in the literature. McHugh et al. found mixed findings in studies focused on dropout from pharmacological and psychological therapies for opioid-dependent patients, indicating the complexity of factors influencing retention [21]. In another study, a secondary analysis involving 1,267 opioid-dependent individuals in treatment programs found that younger age and higher education were significantly associated with extended treatment retention [22]. Another study by Welsh et al. highlighted age-related disparities in treatment admissions for adolescents and young adults with opioid use disorder [23]. This research underscored the differences in treatment approaches and retention rates across different age groups, suggesting that younger individuals might have distinct needs and challenges in opioid use disorder treatment​. This observation can be attributed to a confluence of factors. Younger individuals often exhibit greater adaptability and resilience, which can serve as catalysts for a smoother transition into a drug-free life. Their relative youth may also confer a reduced burden of health complications stemming from prolonged opioid use, thereby rendering the recovery process less arduous. Additionally, the presence of external motivators, such as familial and career aspirations, may fortify their commitment to the recovery journey. Also, individuals presenting at a younger age may exhibit less severe dependence and less socioeconomic impairment, rendering them more likely to continue with treatment.

Furthermore, the research underscores the pivotal role of education in predicting improved follow-up outcomes for individuals grappling with opioid addiction. Education equips individuals with a deeper comprehension of the consequences of addiction and enhances their capacity to make informed decisions regarding treatment options. Higher educational attainment also facilitates greater access to mental health services and fortifies support networks, both of which are instrumental in guiding individuals along the path to recovery.

The study also found that subjects belonging to nuclear families exhibited longer treatment retention. The study emphasizes the vital role of being part of a nuclear family in predicting more favorable follow-up results. It suggests that the stability found in nuclear family settings, marked by strong interpersonal bonds and dependable emotional backing, can be a substantial advantage for individuals in their journey toward recovery. Such families may offer a structured and nurturing environment that helps reduce the likelihood of relapse and reinforces the determination to maintain sobriety [24,25,26].

Notably, other sociodemographic characteristics such as family income, gender, residential status, marital status, family type, living arrangement, employment status, socioeconomic status, and HIV status did not yield significant differences in treatment retention. In a study by Perreault et al., age, sex, and psychological distress were not significantly related to treatment retention [27]. According to a study by Mullen et al., men are more prone to discontinue treatment [28].

Statistical analysis using the one-way ANOVA test for significance identified a shorter duration of opioid intake as being associated with longer treatment retention, potentially indicating a less severe form of dependence. This underscores the paramount importance of early intervention and the accessibility of treatment options. Initiating help-seeking behaviors and embarking on the recovery journey at an earlier stage may yield fewer physical and psychological complications, ultimately facilitating enduring recovery [29].

However, no significant disparities were observed in age at first use or route of intake between the two groups. In a study by Shouan et al., no significant difference was observed in any of the demographic and clinical profiles of the groups that followed up and those who dropped out except for the route of drug use; non-intravenous drug use was associated with dropping out [30].

The significance of social support systems in treatment retention was underscored by the discovery that enhanced social relationships, as measured by the social relationship domain score of QOL, predicted prolonged treatment retention. Thus, the study places substantial emphasis on the role of robust social support systems in sustaining recovery efforts. Whether emanating from family, friends, support groups, or healthcare professionals, social support plays a pivotal role. A network of individuals who comprehend the challenges of recovery and offer unwavering emotional, practical, and motivational support significantly enhances the prospects of a successful recovery.

The study has several limitations, including a small sample size that restricts its applicability to the general population. It was conducted at a single site, limiting its scope. Future research, preferably multicentric with larger sample sizes, is recommended. Addiction is a pervasive chronic illness impacting all aspects of life, and longer-term observations can provide valuable insights. The study should also assess dimensions like risk-taking behavior, legal issues, and financial losses during OST treatment. Future research should compare community-based and clinic-based OST delivery in resource-constrained settings.

## Conclusions

This study investigates the intricate determinants influencing success in opioid addiction recovery. Younger age and higher education emerge as key predictors, providing a deeper understanding of addiction consequences and access to support. Belonging to a nuclear family fosters stability and emotional support, aiding recovery. Early intervention is crucial, as shorter addiction durations lead to better outcomes. Robust social support, whether from family, friends, or professionals, significantly aids recovery. Overall, the study unveils the interconnected role of age, education, family, addiction duration, and support systems in shaping recovery from opioid addiction. This adds to the findings from Western countries where mixed findings are reported about these factors.

These profound insights should serve as invaluable guidance for healthcare professionals and policymakers as they endeavor to craft more effective, targeted interventions to bolster those grappling with opioid addiction. Ultimately, these efforts aim to enhance the effectiveness of addiction treatment programs and ameliorate the overall well-being of individuals afflicted by opioid dependence.
